# Interleukin‐6 in the central amygdala is bioactive and co‐localised with glucagon‐like peptide‐1 receptor

**DOI:** 10.1111/jne.12722

**Published:** 2019-05-23

**Authors:** Fredrik Anesten, Adrià Dalmau Gasull, Jennifer E. Richard, Imre Farkas, Devesh Mishra, Lilly Taing, Fuping Zhang, Matti Poutanen, Vilborg Palsdottir, Zsolt Liposits, Karolina P. Skibicka, John‐Olov Jansson

**Affiliations:** ^1^ Department of Physiology Institute of Neuroscience and Physiology The Sahlgrenska Academy at the University of Gothenburg Gothenburg Sweden; ^2^ Wallenberg Centre for Molecular and Translational Medicine Gothenburg Sweden; ^3^ Department of Neuroscience Faculty of Information Technology and Bionics Pázmány Péter Catholic University Budapest Hungary; ^4^ Laboratory of Reproductive Neurobiology Institute of Experimental Medicine Hungarian Academy of Sciences Budapest Hungary; ^5^ Institute of Biomedicine Research Centre for Integrative Physiology and Pharmacology, and Turku Center for Disease Modeling University of Turku Turku Finland

**Keywords:** amygdala, GLP‐1, immunohistochemistry, interleukin‐6, obesity

## Abstract

Neuronal circuits involving the central amygdala (CeA) are gaining prominence as important centres for regulation of metabolic functions. As a part of the subcortical food motivation circuitry, CeA is associated with food motivation and hunger. We have previously shown that interleukin (IL)‐6 can act as a downstream mediator of the metabolic effects of glucagon‐like peptide‐1 (GLP‐1) receptor (R) stimulation in the brain, although the sites of these effects are largely unknown. In the present study, we used the newly generated and validated RedIL6 reporter mouse strain to investigate the presence of IL‐6 in the CeA, as well as possible interactions between IL‐6 and GLP‐1 in this nucleus. IL‐6 was present in the CeA, mostly in cells in the medial and lateral parts of this structure, and a majority of IL‐6‐containing cells also co‐expressed GLP‐1R. Triple staining showed GLP‐1 containing fibres co‐staining with synaptophysin close to or overlapping with IL‐6 containing cells. GLP‐1R stimulation enhanced IL‐6 mRNA levels. IL‐6 receptor‐alpha (IL‐6Rα) was found to a large part in neuronal CeA cells. Using electrophysiology, we determined that cells with neuronal properties in the CeA could be rapidly stimulated by IL‐6 administration in vitro. Moreover, microinjections of IL‐6 into the CeA could slightly reduce food intake in vivo in overnight fasted rats. In conclusion, IL‐6 containing cells in the CeA express GLP‐1R, are close to GLP‐1‐containing synapses, and demonstrate increased IL‐6 mRNA in response to GLP‐1R agonist treatment. IL‐6, in turn, exerts biological effects in the CeA, possibly via IL‐6Rα present in this nucleus.

## 1 INTRODUCTION

Interleukin‐6 (IL‐6) is a cytokine with a large variety of functions in disease and health.^1^ Released together with other classic pro‐inflammatory cytokines, such as tumour necrosis factor (TNF)‐α, IL‐6 appears to have deleterious effects on metabolism.^2^, ^3^, ^4^ By contrast, when IL‐6 is released together with metabolically beneficial hormones such as leptin, glucagon‐like peptide‐1 (GLP‐1) and amylin, IL‐6 appears to have positive effects on metabolism.^5^, ^6^, ^7^, ^8^, ^9^ IL‐6 is not only produced in adipose tissue, in skeletal muscle during exercise and in immune cells, but also is abundant in the central nervous system (CNS).^1^, ^5^, ^10^, ^11^, ^12^ The membrane bound IL‐6 receptor, IL‐6 receptor‐alpha (IL‐6Rα) is expressed in a major portion of fundamental energy‐regulatory hypothalamic nuclei in the healthy mouse brain, mainly in neurones, thus forming an anatomical basis for the local effects of IL‐6.^13^, ^14^, ^15^, ^16^

IL‐6^−/−^ mice develop mature‐onset obesity at around 6 months of age.^17^ Intracerebroventricular administration (but not peripheral administration) of IL‐6 decreases body fat in rodents, suggesting that the anti‐obesity effect of IL‐6 is exerted at the level of the CNS.^18^, ^19^ Central IL‐6 has been shown to play a role in the anti‐obesity effects of GLP‐1 analogues, such as exendin‐4 (Ex‐4), mainly via reduced food intake.^20^, ^21^ In the hypothalamus and hindbrain, IL‐6 appears to act as a downstream mediator for the effects of GLP‐1R‐stimulation,^21^ as well as amylin action.^5^ Thus, IL‐6 may act as a regulator of anorexia and body weight in healthy animals. In addition, the cachexia observed in various diseases appears to depend partially on IL‐6 release.^2^, ^22^, ^23^ In mouse cancer models, blocking of IL‐6 synthesis has been shown to attenuate cachexia progression.^24^

GLP‐1, a post‐translational proglucagon product, is a neuropeptide produced peripherally in ileal L‐cells of the intestine, as well as centrally in preproglucagon (PPG) neurones of the nucleus of the solitary tract (NTS).^25^ GLP‐1 production causes an increase of insulin release from pancreatic β‐cells and a decrease of glucagon release from pancreatic α‐cells. GLP‐1 has a very short half‐life in serum because it is rapidly degraded by the enzyme dipeptidyl‐peptidase 4. However, long acting GLP‐1 analogues used to treat obesity and type 2 diabetes, such as Ex‐4, are less easily degraded.^26^, ^27^

GLP‐1 and the ability of its analogues to increase insulin secretion and control blood glucose levels, as well as their anti‐obesity effects, have been widely explored.^20^, ^28^, ^29^, ^30^ However, the potential for IL‐6 to act as a downstream signalling peptide of GLP‐1 has not yet been fully explored. It has been observed that GLP‐1 analogues decrease food intake as well as body weight. Previous studies have reported that these anorexic effects are exerted at the level of the CNS^27^, ^31^, ^32^ and it has been suggested that GLP‐1 may act on POMC/CART expressing neurones in the arcuate nucleus of the hypothalamus.^33^ Moreover, peripheral GLP‐1 and its analogues have been reported to cross the blood‐brain barrier, making the central GLP‐1 receptor (GLP‐1R) a possible pharmaceutical target.^34^, ^35^

GLP‐1Rs are highly expressed in energy‐balance‐regulating areas, such as the hypothalamus, hindbrain and amygdala.^36^ It is likely that the principal source of ligand to these receptors is the GLP‐1‐producing‐neurones of the NTS that produce PPG, a precursor for e g GLP‐1. Furthermore, it has been shown that PPG‐projections reach, among other nuclei, the central amygdala (CeA).^28^ The CeA is involved in fear‐, stress‐ and drug‐related responses.^37^ Aside from these functions, the CeA was implied to be important in regulation of food intake and energy expenditure.^38^ Bilateral lesions of the amygdala in rats induce hyperphagia and weight gain, further supporting its role as an anorexigenic nucleus.^39^

Studies of the amygdala have shown that PPG‐projections lie in proximity to the same neurones, mainly in the capsular part of the CeA. Moreover, cells in both the CeA and medial amygdala display GLP‐1R immunoreactivity.^20^, ^40^ Previous studies have shown that GLP‐1R stimulation increases neural activity in the CeA, which leads to a decrease in food consumption in rats,^38^ although the regulating mechanism remains unclarified. Thus, there are plenty of data in the literature suggesting that GLP‐1 exerts an anorectic effect in CeA.^38^ In the present study, we investigated the effects exerted on CeA by IL‐6, another peptide with well‐known anorectic and fat mass suppressing effects in the CNS according to most (but not all) studies.^17^, ^19^, ^21^, ^41^ Moreover, we investigated the possible interactions between IL‐6 and GLP‐1, two peptides shown to interact in the brain in regulation of metabolic functions.^21^

## 2 MATERIALS AND METHODS

### 2.1 Animals

Twelve‐week‐old male and female RedIL6 strain mice were used to visualise IL‐6. Full details on this strain have been provided previously.^42^

Eight‐week‐old male and female GLP‐1R reporter mice were kindly provided by Professor Stefan Trapp, Centre for Cardiovascular and Metabolic Neuroscience, University College of London, UK. Aditional data on this strain have been provided previously.^40^

Twelve‐week‐old male and female PPG‐reporter (Venus) mice were kindly provided by Professors Fiona M. Gribble and Frank Reimann, Institute of Metabolic Science & MRC Metabolic Diseases Unit, University of Cambridge, UK.^43^

For intra‐amygdala injections and electrophysiology, 7‐8‐week‐old male Sprague‐Dawley rats were used.

Animals had free access to water and standard chow pellets (Tekland Global, Harlan, The Netherlands) and were kept under a 12:12 hour light/dark cycle (lights on 6.00 am) at 24‐26°C and 50%‐60% relative humidity, with food available ad lib., unless otherwise specified. The local ethics committee for animal care at the University of Gothenburg approved all of the animal procedures.

### 2.2 Tissue preparation for immunohistochemistry

Mice were deeply anaesthetised and perfused transcardially with heparinised saline (50 IU mL^‐1^) followed by 4% paraformaldehyde in 0.1 mol L^‐1^ phosphate buffer. The brains were removed and post‐fixed in 4% paraformaldehyde in 0.1 mol L^‐1^ phosphate buffer containing 15% sucrose overnight at 4°C. Next, they were transferred to a 30% sucrose solution in 0.1 mol L^‐1^ phosphate buffer until sectioning. Coronal 20‐μm thick serial sections of the amygdala were cut using a CM3050S cryostat (Leica Microsystems, Wetzlar, Germany) and stored in cryoprotectant solution (25% ethylene glycol; 25% glycerol; 0.05 mol L^‐1^ phosphate buffer). For GFP/GLP‐1 co‐staining, we instead used 30‐μm sections that were not post‐fixed in 4% paraformaldehyde in 0.1 mol L^‐1^ phosphate buffer containing 15% sucrose overnight. Coronal sections corresponding to bregma −1.07 to −1.55, interneural 2.24 to 2.72^44^ were selected for staining.

### 2.3 Immunohistochemistry

**Figure 1:**
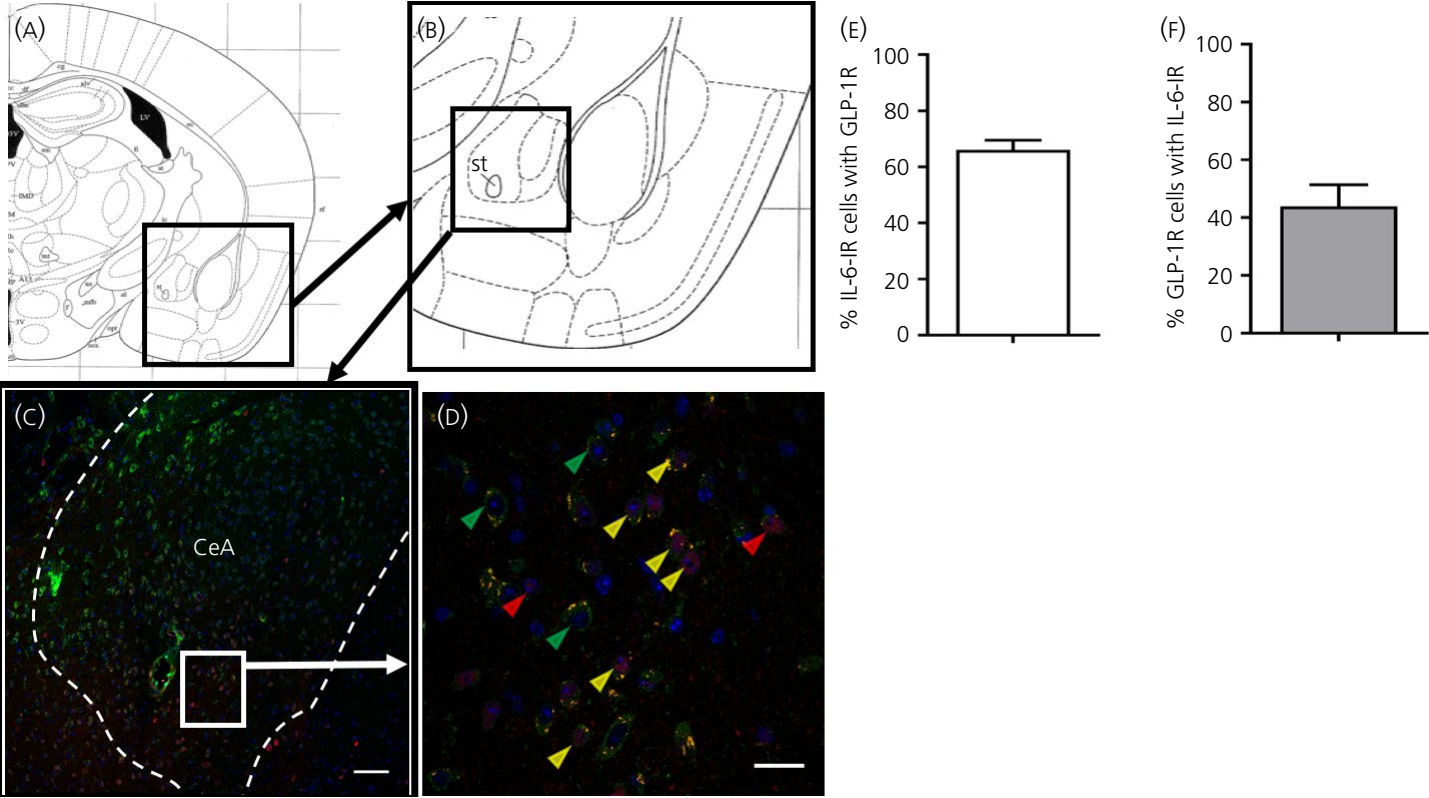
Glucagon‐like peptide‐1 receptors (GLP‐1R) are present in interleukin (IL)‐6 containing cells in the central amygdala. A, B, Anatomical overview images showing the region from which the immunohistochemistry magnifications were taken. C, D, Strong overlap between IL‐6 immunoreactivity (red) and GLP‐1R reporter mouse fluorescence (green). Cell nuclei are stained with 4′,6‐diamidino‐2‐phenylindole (blue). Yellow arrowheads show examples of cells where GLP‐1R and IL‐6 co‐localised, whereas red and green arrowheads show examples of cells with only IL‐6 or GLP‐1R fluorescence, respectively The area in (D) is a magnification of the area marked in (C). E, F, Sixty‐six percent of IL‐6‐immunoreactive (‐IR) cells also displayed GLP‐1R‐fluorescence (E), whereas approximately 44% of GLP‐1R‐fluorescent cells also showed IL‐6 immunoreactivity (F). Scale bars: overview = 80 μm, zoom 10 μm. CeA, central amygdala

Briefly, sections were rinsed in wash buffer (0.1 mol L^‐1^ TrisHCl, pH 7.5, 0.15 mol L^‐1^ NaCl) and blocked for 1 hour with TNB with 0.2% Triton‐X‐100 (Perkin Elmer, Waltham, MA, USA). Sections were incubated with primary antibodies (Table 1) overnight at 4°C. After rinsing, sections were incubated for 1 hour with secondary antibodies (Table 1) diluted in TNB blocking reagent 0.2% Triton‐X‐100 (Perkin Elmer, Waltham, MA, USA). Sections were rinsed and stained with appropriate secondary antibodies. After further washing, cell nuclei were stained with 4’,6‐diamidino‐2‐phenylindole (DAPI) (dilution 1:5000, D1306; Thermo Fisher Scientific, Waltham, MA, USA) for 5 minutes, rinsed and mounted in mounting medium containing prolong diamond anti‐fade (P36965; Thermo Fisher Scientific). As a control for the secondary antibodies, some sections were incubated with mismatching primary and secondary antibodies, resulting in negative staining, as a control for unwanted cross‐reactivity. Primary and secondary antibodies, dilutions used and catalogue numbers, as well as the manufacturers providing them, are listed in Table 1. Cartoons in Figures 1A,B and 3A,B are adapted from Paxinos and Franklin.^44^ For IL‐6 staining in GLP‐1R and GLP‐1 (Venus) reporter mice, a previously validated IL‐6 antibody^42^, ^45^ was used. Confirmation that this antibody shows markedly less staining also in the amygdala of RedIL6 homozygous mice is provided in the Supporting information (Figure S1).

**Table 1:** List of antibodies used in the present study

Antiserum	Dilution	Catalogue number	Manufacturer
Mouse anti‐RFP Tag	1:200	MA5‐15257	Thermo Fisher Scientific, Waltham, MA, USA
Rabbit anti‐RFP	1:200	R10367	Life Technologies Europe, Stockholm, Sweden
Chicken anti‐NeuN	1:200	Ab134014	Abcam, Cambridge, UK
Rabbit anti‐IL‐6	1:200	1265‐R	Santa Cruz Biotechnology, Santa Cruz, CA, USA
Rat anti‐IL‐6Rα	1:20	BAM18301	R&D Systems, Minneapolis, MN, USA
Mouse anti‐Synaptophysin	1:200	Ab8049	Abcam, Cambridge, UK
Goat anti‐chicken Alexa fluor 488	1:250	A‐11042	Molecular Probes, Carlsbad, CA, USA
Goat anti‐rabbit Alexa fluor 488	1:250	A‐11008	Molecular Probes, Carlsbad, CA, USA
Goat anti‐rabbit Alexa fluor 568	1:250	A‐11036	Molecular Probes, Carlsbad, CA, USA
Donkey anti‐goat Alexa 488	1:250	A‐11055	Molecular Probes, Carlsbad, CA, USA
Goat anti‐rabbit Alexa 350	1:250	A‐11046	Molecular Probes, Carlsbad, CA, USA
Chicken anti‐mouse 488	1:250	A‐21200	Molecular Probes, Carlsbad, CA, USA

### 2.4 Confocal microscopy and cell counting

Images of the stained sections were obtained using either a confocal microscope system (LSM 700; Carl Zeiss, Oberkochen, Germany), together with a Plan APO ×40 A/1.40 oil lens (Nikon, Tokyo, Japan) (for close‐up pictures) or a Plan Fluor ×20/0.75 lens (for anatomical overview pictures) and a solid‐state laser. For co‐localisation, focus stacking was used to achieve a greater depth of field. Micrographs were adjusted for brightness and contrast in fiji 46 and overview slides were constructed using a fiji plug‐in.^47^

Co‐localisation was quantified from 20‐μm sections from four separate slices from mouse brains per experiment. Double or triple channel confocal images (depending on the number of fluorophores used) covering the entire CeA were generated with a Plan Fluor ×20/0.75 lens and a solid‐state laser. A tile scan of 3 × 3 tiles was obtained from the centre of the CeA, covering the entirety of the nucleus. Cells were considered labelled when the staining was clearly above background, as measured by fluorescence intensity measurements. The emission spectrum of the secondary fluorescent antibody is well known. By adjusting the beam splitter of the confocal microscope, the signal of the fluorophore was maximised at the same time as minimising background fluorescence. Cells were only considered labelled if their nucleus was in the focal plane. Four mice per treatment group and experiment were used for immunohistochemistry. Cell counting was performed from one brain slice from each of the four animals. Representative confocal micrographs from these animals were used to construct images. Student's *t* test was used to determine statistical significance of the differences in co‐localisation.

### 2.5 Electrophysiology

Rats were anaesthetised using isoflurane inhalation. The brain was removed rapidly and immersed in ice‐cold sodium‐free solution.^22^ Acute 300‐μm‐thick coronal slices containing the CeA were prepared with a VT‐1000S vibratome (Leica) in the sodium‐free solution and then equilibrated in normal artificial cerebral spinal fluid (aCSF) (135 mmol L^‐1^ NaCl, 3.5 mmol L^‐1^ KCl,26 mmol L^‐1^ NaHCO_3_, 1.2 mmol L^‐1^ MgSO_4_, 1.25 mmol L^‐1^ NaH_2_PO_4_, 2.5 mmol L^‐1^ CaCl_2_ and 10 mmol L^‐1^ glucose, bubbled with O_2_/CO_2_). Loose‐patch clamp measurements to record action currents were carried out as described previously^23^ with slight modifications. Briefly, pipette potential was held at 0 mV, pipette resistance 1‐2 MΩ and resistance of loose‐patch seal 7‐40 MΩ. The pipette solution contained: 123 mmol L^‐1^ NaCl, 3.5 mmol L^‐1^ KCl, 2.5 mmol L^‐1^ CaCl_2_, 1.3 mmol L^‐1^ MgCl_2_, 10 mmol L^‐1^ Hepes and 10 mmol L^‐1^ glucose (pH 7.3; with NaOH). CeA was identified under microscopic control. Measurements were carried out with an initial control recording (4 minutes); then, in the first experimental group of neurones, IL‐6 (1 nmol L^‐1^) was added to the normal aCSF by a single bolus into the recording chamber, and the recording continued for a subsequent 11 minutes. In a second experimental group of neurones, a cocktail of IL‐6 and a IL‐6 neutralising antibody (1 mol L^‐1^; Tocris Bioscience, St Louis, MO, USA) was applied after the initial recording of basal firing. Each neurone served as its own control when drug effects were evaluated.

### 2.6 RNA isolation and mRNA expression

CeA gene expression levels were measured after lateral ventricle injection of Ex‐4 (1933; RnD Systems Inc [3 mg kg^‐1^]) or vehicle (aCSF) in 16 mice (8 + 8). Ninety minutes after Ex‐4 or aCSF injection, the brains were rapidly removed and the CeA was dissected using a brain matrix, frozen in liquid nitrogen and stored at −80°C. Individual brain samples were homogenised in Qiazol (Qiagen, Valancia, CA, USA) using a TissueLyzer (Qiagen). Total RNA was extracted using RNeasy Lipid Tissue Mini Kit (Qiagen) with additional DNAse treatment (Qiagen). RNA quality and quantity were assessed by spectrophotometric measurements (Nanodrop 1000; NanoDrop, Wilmington, DE, USA). For cDNA synthesis, an iScript cDNA Synthesis kit (Bio‐Rad, Hercules, CA, USA) was used. Real‐time RT PCR was performed using TaqMan® probe and primer sets for target genes chosen from an on‐line catalogue (Actb‐Mm00607939_s1, IL6‐Mm00446190_m1; Applied Biosystems, Foster City, CA, USA). Gene expression values were calculated based on the *C*_t_ method,^48^ where the vehicle‐injected group was designated as the calibrator. Beta actin was used as reference gene.

### 2.7 In situ hybridisation

**Figure 2:**
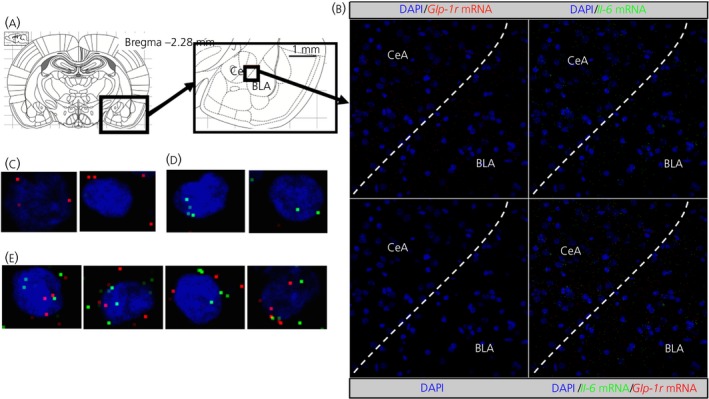
Glucagon‐like peptide‐1 receptors (GLP‐1R) and interleukin (IL)‐6 mRNA is partially co‐localised in the central amygdala. GLP‐1R (red) and IL‐6 (green) mRNA was found partially in the same cells in the central amygdala (CeA). Anatomical overview images (A) showing the region from which the magnifications were taken. Representative images (B) of the region show partial co‐expression of GLP‐1R and IL‐6 in CeA cells. Magnified images show cells expressing only GLP‐1R mRNA (C), only IL‐6 mRNA (D) or co‐expression (E). BLA, basolateral amygdala; DAPI, 4′,6‐diamidino‐2‐phenylindole

For RNAscope® (ACD, Newark, CA, USA) investigations, central amygdala containing brain sections (12 μm thick) were cut and fixed in 10% formalin (Thermo Fisher Scientific) for 30 minutes. Following two quick washes in phosphate‐buffered saline, brain slices were dehydrated in 50% (5 minutes), 70% (5 minutes) and twice in 100% (5 minutes each) ethanol and then pretreated with protease solution at room temperature for 30 minutes. The protease was washed away with phosphate‐buffered saline. Target probe for GLP‐1R (Rn‐GLP‐1R 315221‐C1), IL‐6 (Rn‐IL‐6‐C3 427141‐C3) and negative control probes were applied directly on the sections to cover them completely and incubated at 40°C for 2 hours in a HybEZ oven (ACD). Then, preamplifier and amplifier probes were added (AMP1, 40°C for 30 minutes; AMP2, 40°C for 15 minutes; AMP3, 40°C for 30 minutes; AMP4‐Alt C for 15 minutes). Finally, brain sections were incubated for 30 seconds with DAPI and mounting medium for fluorescence (Vectashield; Vector Laboratories, Inc., Burlingame, CA, USA). Fluorescence images of the central amygdala were captured at 40× using a LSM700 Zeiss confocal microscope and images were processed using zen lite (Carl Zeiss). The cartoon in Figure 2A is adapted from Paxinos and Watson.^49^

### 2.8 Intra‐amygdala IL‐6 administration and food intake

Rats were implanted with a guide cannula targeting the CeA (26‐gauge; Plastics One Inc., Roanoke, VA, USA) under ketamine anaesthesia as described previously.^50^, ^51^ The coordinates chosen for the CeA were: ±4.2 mm from the midline and −2.8 mm posterior to bregma, with injector aimed 8.6 mm ventral to skull. Rats were allowed a minimum of 7 days of rest after surgery.

To test the effects of IL‐6, rats were injected with IL‐6 (0.2 or 1 μg per 0.5 μL) or vehicle (aCSF). Rats were food‐restricted to 50% of their normal intake overnight. Chow intake was measured 1, 3 and 6 hours after injection. Each treatment was counterbalanced, where each condition was separated by a minimum of 2 days.

### 2.9 Statistical analysis

Graphs were constructed using prism, version 8 (GraphPad Software Inc., San Diego, CA, USA). Statistical analyses were conducted using Students *t* test. *P* < 0.05 was considered statistically significant.

## 3 RESULTS

### 3.1 GLP‐1R and IL‐6 overlap on protein and mRNA levels in the CeA

GLP‐1R‐fluorescent cells were present in the CeA, mainly in the lateral and medial parts, whereas IL‐6 immunoreactivity was more widely spread in the entirety of the nucleus (Figure 1C). Magnified views of the CeA showed cells with IL‐6 (red), GLP‐1R (green) and co‐staining of IL‐6 and GLP‐1R (Figure 1D).

**Figure 3:**
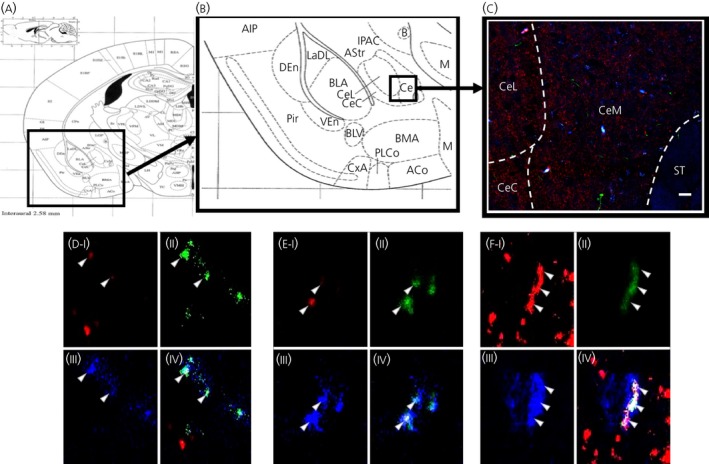
Some glucagon‐like peptide‐1 (GLP‐1) fibres form synapses with interleukin (IL)‐6‐immunoreactive cells in the central amygdala (CeA). GLP‐1 (green), synaptophysin (red) and IL‐6 (blue) was found to co‐localise in some structures of the CeA. Anatomical overview images (A, B) showing the region from which the magnifications were taken. Representative image (C) of the region showing partial co‐localisation of GLP‐1, IL‐6 and synaptophysin. Triple‐channel confocal micrographs (D‐F) showing micrographs of single cells with synaptophysin (I), GLP‐1 (II), IL‐6 (III) and merged channels (IV). CeL, lateral central amygdala; CeM, medial central amygdala; CeC, capsular central amygdala; ST, striatum. Scale bars: overview = 80 μm. Pir ‐ Piriform cortex, AIP ‐ Agranulos insular cx, posterior, DEn ‐dors endopiriform nucleus, LaDL ‐ lateral amygdalar nucleus, dorsolateral, IPAC ‐ Interstitial nucleus, p limb, ac, AStr ‐ Amygstriat transit area, BLA ‐ Basolateral amygdala, B ‐ Basolateral nucleus, VEn ‐ Ventral endopirifom nucleus, BLV ‐ Basolateral amygdalar nucleus, ventral, PLCo ‐ Postlateal cx amygdalar nucleus, CxA ‐ Cortex‐amygdala transit zone, ACo ‐ Anterior cortical amygdalar nucleus

Cell counting showed that 66% of the IL‐6‐immunoreactive cells were also GLP‐1R‐fluorescent, whereas 44% of GLP‐1R‐fluorescent cells were also IL‐6‐immunoreactive (Figure 1E,F). In line with this, many cells in the CeA expressing GLP‐1R mRNA also expressed IL‐6 mRNA, as indicated by fluorescent in situ hybridisation (RNAscope®) (Figure 2). Examples of cells that expressed both IL‐6 and GLP‐1R are shown in Figure 2E. Quantification of 124 DAPI stained nuclei showed 86% co‐localisation of GLP‐1R+ cells with IL‐6+, and 79% co‐localisation of IL‐6+ cells with GLP‐1R+. Using the novel RedIL6 reporter mouse strain, we found a pattern of fluorescence similar to that with the anti‐IL‐6 antibody staining (Figure 3A,B).

### 3.2 GLP‐1 is present in synapses close to IL‐6 fluorescent cells

**Figure 4:**
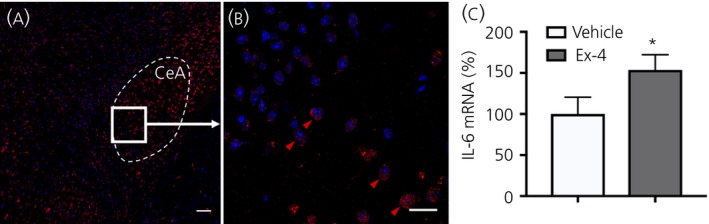
RedIL6 fluorescence is present in the central amygdala (CeA) and i.c.v. administration of exendin‐4 (Ex‐4) increased interleukin (IL)‐6 mRNA expression in the CeA. A, B, Cells in the CeA that contain IL‐6, as indicated by RedIL6 reporter mouse (red), after i.p. infusion of vehicle. Shown is a representative overview of the CeA (A) and magnification (B) of the indicated parts of the CeA in (A). Red arrowheads indicate examples of IL‐6 fluorescent cells (B). Scale bars: overview = 80 μm, zoom = 10 μm. C, The results of a reverse transcriptase‐polymerase chain reaction from 8 + 8 mice showed a 1.5‐fold increase in IL‐6 mRNA after i.c.v. infusion of Ex‐4 compared to infusion of vehicle (artificial cerebrospinal fluid). *P < 0.05 and **P < 0.01, respectively

PPG‐reporter (Venus) mice were co‐stained with IL‐6 and synaptophysin antibodies to determine whether PPG‐synapses were present near IL‐6 cells in the CeA (Figure 4). We found examples of cells where triple co‐localisation was present, suggesting that some PPG‐fibres formed synapses with IL‐6‐immunoreactive cells (Figure 4D‐F).

### 3.3 Central Ex‐4 administration increases IL‐6 mRNA expression in the CeA

Administration of Ex‐4 (3 mg kg^‐1^) to the lateral ventricle of mice resulted in a statistically significant 50% increase in IL‐6 mRNA‐expression after 90 minutes compared to vehicle (aCSF) controls (*P* = 0.04) (Figure 3C).

### 3.4 IL‐6Rα is present in NeuN‐immunoreactive cells in the central amygdala and IL‐6 stimulates the firing rate of neurones in the capsular part of the CeA

**Figure 5:**
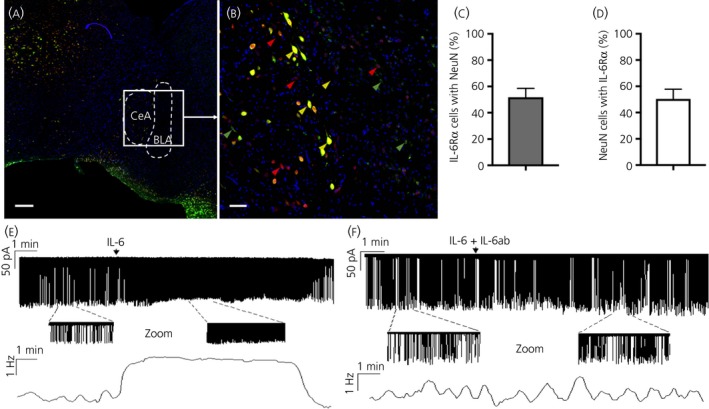
IL‐6 receptor‐alpha (IL‐6Rα) was present in NeuN‐positive cells in the central amygdala and IL‐6 stimulated the firing rate of neurones residing in the capsular part of the central amygdala. IL‐6Rα immunoreactivity (green) and NeuN immunoreactivity (red) partially co‐localised in the central amygdala (CeA). Cell nuclei were stained with 4′,6‐diamidino‐2‐phenylindole (blue). Yellow arrowheads show examples of cells where IL‐6Rα and NeuN immunoreactivity co‐localised, whereas green and red arrowheads show examples of cells with only IL‐6Rα and NeuN immunoreactivity, respectively (A, B). Approximately 50% of IL‐6Rα‐immunoreactive cells also showed NeuN immunoreactivity. Conversely, approximately 50% of NeuN immunoreactive cells also showed IL‐6Rα immunoreactivity (C, D). These results indicate that a substantial proportion of the neurones in the CeA could be responsive to IL‐6. Loose‐patch recording showed that IL‐6 (1 nmol L^‐1^) could rapidly increase the firing rate of the targeted neurones. The zoomed periods and frequency distribution graph under the recording also show this profound elevation (E). A cocktail of IL‐6 and its neutralising antibody (IL‐6 ab) evoked no significant change in the firing rate (F). Arrows show the onset of administration of IL‐6 or the IL‐6 + IL‐6ab cocktail. Scale bars: overview = 80 μm, zoom = 10 μm. BLA, basolateral amygdala

IL‐6Rα antibody was co‐stained with the neurone marker NeuN. We found co‐localisation between the two antibodies in the CeA, mainly in the lateral part (Figure 5A,B). Cell counting showed that approximately 50% of IL‐6Rα‐immunoreactive cells were also NeuN‐immunoreactive (Figure 5). Conversely, approximately 50% of the NeuN‐immunoreactive cells were also IL‐6Rα‐immunoreactive (Figure 5C).

Given that we found IL‐6 and IL‐6Rα in the CeA, we next set out to investigate whether IL‐6 could exert effects in CeA (ie, if the IL‐6Rα found there were functional). Accordingly, IL‐6 was added to neurones of the CeA of acute rat brain slices. Action current firing was then recorded by loose‐patch method. A control period of 5 minutes at the start of the recordings showed that most of these cells fired spontaneously in bursts. The burst frequency was 0.15 ± 0.10 Hz, the number of spikes in a burst was 12 ± 6 and the intraburst frequency of the action current spikes was 6.6 ± 2.34 Hz.

Application of a single bolus of IL‐6 (1 nmol L^‐1^) rapidly increased the firing rate up to 356 ± 48% of the baseline frequency (2.09 ± 0.43 Hz; 12 neurones/12 brain slices/five rats) (Figure 5E) and this significant (*P* < 0.05, Student's *t* test) elevation was mostly because the decrease in the time between bursts resulted in a high‐frequency tonic firing. The effect of IL‐6 lasted for approximately 10 minutes (Figure 5E). Repeated administration of IL‐6 approximately 5 minutes after returning of the frequency to its baseline level resulted in no change. These data are in accordance with the results of Fischer et al^52^ demonstrating that IL‐6 has rendered cells insensitive to further administration of IL‐6 for several hours. In a second experiment, IL‐6 was pre‐incubated with an IL‐6 neutralising antibody for 30 minutes, followed by administration of this cocktail to CeA slices. This resulted in a significant dampening of the effect induced by IL‐6 only on the mean firing rate (160 ± 35%; *P* < 0.05; 10 neurones/10 slices/four rats) (Figure 5F).

### 3.5 IL‐6 administration to CeA slightly reduces 1‐hour chow intake in overnight‐restricted rats

**Figure 6:**
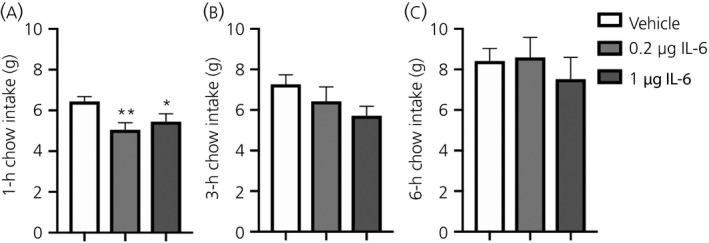
Interleukin (IL)‐6 administration to central amygdala slightly reduced 1‐h chow intake in overnight fasted rats. Chow intake is slightly reduced after 1 h in food‐restricted rats after intra‐amygdalar administration of 0.2 or 1 μg of IL‐6 or vehicle to the central amygdala. No effect on chow intake is seen 3 h (B) or 6 h (C) after injection

Administration of 0.2 or 1 μg (Figure 6) of IL‐6 to the CeA in rats slightly decreased 1‐hour chow intake (Figure 6A).

By contrast to the 1‐hour time point, there was no effect on 3‐hour (Figure 6B) or 6‐hour (Figure 6C) chow intake after IL‐6 administration. The reduction of food intake was seen in animals that had been fasted overnight, whereas there was no effect in ad lib. fed animals (not shown). Administration of an IL‐6/CGRP cocktail did not potentiate the suppression of food intake seen in IL‐6 treated and food‐restricted rats (not shown).

## 4 DISCUSSION

### 4.1 Potential interactions between GLP‐1 and IL‐6 in the CeA

Formerly, the amygdala has been seen mainly as a nucleus important for fear‐ and stress conditioning.^37^ However, recently, it has also been implicated as important for the regulation of energy balance, including feeding.^38^, ^53^ In the present study, we aimed to investigate the anatomical basis for possible interactions in the amygdala between IL‐6 and GLP‐1. A newly developed and validated RedIL6 mouse strain was used to map the location of IL‐6 in the CeA. The results obtained showed that a majority (approximately 66%) of IL‐6 expressing cells also displayed GLP‐1R immunoreactivity.

In the present study, we used RedIL6, an IL‐6 reporter mouse that was described recently.^42^ Briefly, the RedI6 reporter mouse was generated according to a gene knock‐in protocol. The red fluorescent reporter mKate2 gene sequence replaced the sequence of the first three exons of the mouse IL‐6 gene. In this strain, endogenous mKATE2 fluorescence was weak and therefore immunohistochemical staining with an anti‐RFP antibody was used to obtain a better signal. The mKATE2 reporter staining reflects IL‐6 expression, as supported by mKATE2 staining being co‐localised with IL‐6 mRNA, which was identified by RNAscope. Moreover, the mKATE2 reporter staining co‐localised with the staining obtained by an anti‐IL‐6 antibody, which, as shown by ourselves (see Supporting information, Figure S1, 42), as well in previous studies,by others^45^ demonstrated less staining in different strains of IL‐6 gene knockout mice

The results from Venus PPG/GLP‐1 reporter mice implicated that there were also GLP‐1 fibres present in the CeA. These GLP‐1 containing fibres are likely to originate in the NTS, which is a major location of GLP‐1 producing neuronal cell bodies.^25^, ^54^ Some of these GLP‐1 containing fibres co‐stained with synaptophysin close to IL‐6‐immunoreactive cell bodies, suggesting that GLP‐1 fibres may synapse with IL‐6‐immunoreactive cells in the CeA and thereby regulate IL‐6 release. The validity of the IL‐6 antibody used for in the present study on immunoreactivity was supported by IL‐6 immunoreactivity being markedly decreased in brains from mice with global IL‐6 knockout.^45^

Previously, we have reported that GLP‐1R agonists can increase the expression of IL‐6 and also that IL‐6 may mediate the decrease in food intake and body fat mass induced by GLP‐1 via actions in the hypothalamus and the brainstem.^21^ In the present study, we show that i.c.v. administration of Ex‐4 resulted in a 50% increase in IL‐6 mRNA in the CeA compared to vehicle after 90 minutes.

Because IL‐6 immunoreactivity was present in GLP‐1R‐fluorescent cells, we speculate that Ex‐4 from the CSF may affect these cells directly. Physiologically, GLP‐1 may arrive at the amygdala via fibres from NTS. Further studies are needed to fully clarify the possible mechanisms of interaction between GLP‐1 and IL‐6 in CeA.

### 4.2 The presence of functional IL‐6Rα in the CeA

IL‐6Rα immunoreactivity was present in over half of the CeA cells with NeuN (a neuronal marker) immunoreactivity. This is consistent with the previously reported co‐localisation of these two antibodies in both the hypothalamus and hindbrain.^13^, ^14^, ^15^, ^16^, ^55^ We next determined whether these receptors were functional. Using electrophysiology, we found that these receptors indeed responded to IL‐6 treatment with an increased firing rate and that this effect could be attenuated by an IL‐6 neutralising antibody.

Further support for functional IL‐6Rα in CeA came from the finding that administration of IL‐6 to the CeA partially inhibited feeding. The latter finding is in line with studies by Timper et al^6^ reporting a similar effect after i.c.v. administration. However, further studies are needed to fully determine whether or not IL‐6 is an important player in amygdala energy balance regulation.

### 4.3 Summary

Taken together, our data indicate that IL‐6 is present in approximately 40% of GLP‐1R cells in the CeA and also that PPG‐fibres appear to synapse with IL‐6‐immunoreactive cells in this nucleus. Moreover, i.c.v. administration of Ex‐4 induced an increase in IL‐6 mRNA in this nucleus. Approximately 50% of the neurones in the CeA expressed the ligand binding receptor IL‐6Rα. The administration of exogenous IL‐6 to CeA brain slices caused electrophysiological stimulation of IL‐6Rα, an effect that is quite rapid and therefore likely to be exerted directly. Moreover, treatment with IL‐6 to CeA of overnight fasted mice reduced food intake, further supporting a biological role for IL‐6Rα in the CeA, although further studies are needed to fully clarify this role.

Several brain areas involved in regulation of energy balance appear to contain IL‐6 ligand (present study, as well as Fredrik, A,Jansson, JO, unpublished results) and IL‐6Rα,^13^, ^14^, ^15^, ^16^, ^56^ which, in some cases, have been shown to be functional, as indicated by their responsiveness to IL‐6 treatment.^13^, ^55^ Deprivation of endogenous IL‐6 by different means results in metabolic effects, including an increased fat mass.^10^, ^17^, ^21^, ^57^ Moreover, the results obtained in several other experimental paradigms are accordance with the effects of IL‐6 in the CNS on energy balance.^58^, ^59^, ^60^ Taken together, these findings are in line with the hypothesis that IL‐6 could act as a neuropeptide regulating energy balance in different parts of the brain.
