# Supplementary material for: Interleukin‐6 in the central amygdala is bioactive and co‐localised with glucagon‐like peptide‐1 receptor
Source: J Neuroendocrinol. 2019 May 23;31(6):e12722. doi: 10.1111/jne.12722 (PMC6618171; doi:10.1111/jne.12722)
Supplement: Supplementary file 1 [file JNE-31-na-s001.docx]

**Supplementary Information**


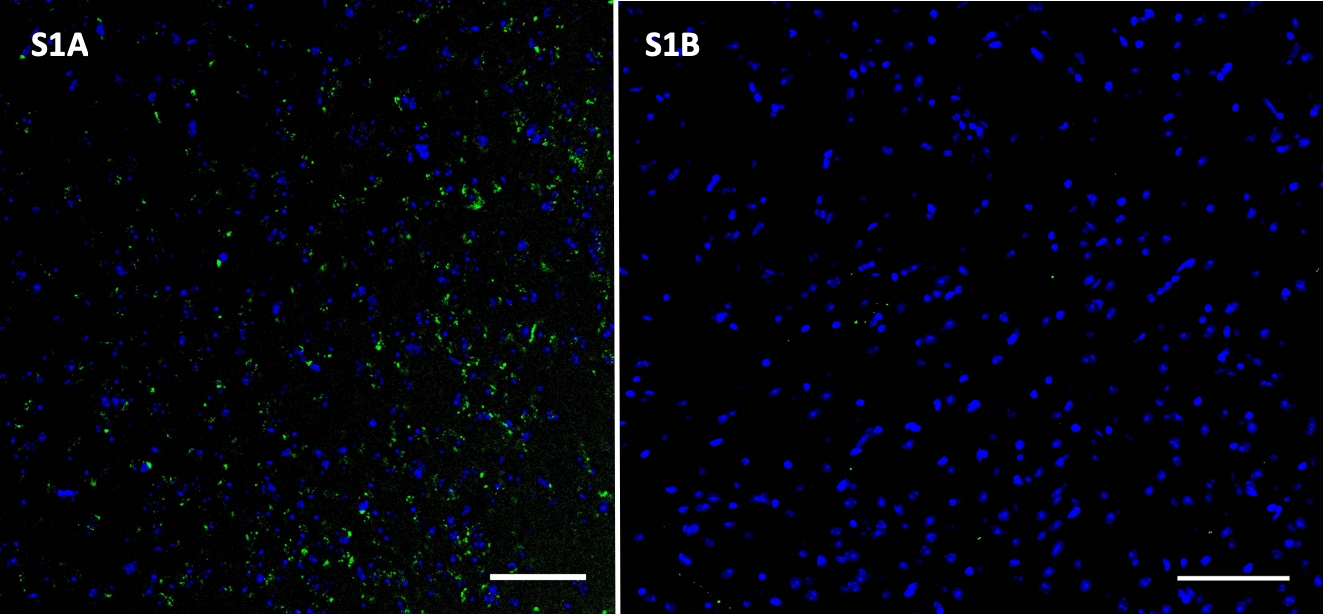


**Fig S1.** Immunohistochemistry of the Central Amygdala showing IL-6 ab immunoreactivity (green) in wt (S1A) or homozygous RedIL6 (S1B) mice.
